# Processing LHC data in the UK

**DOI:** 10.1098/rsta.2012.0094

**Published:** 2013-01-28

**Authors:** D. Colling, D. Britton, J. Gordon, S. Lloyd, A. Doyle, P. Gronbech, J. Coles, A. Sansum, G. Patrick, R. Jones, R. Middleton, D. Kelsey, A. Cass, N. Geddes, P. Clark, L. Barnby

**Affiliations:** 1 Department of Physics, Imperial College London, Blackett Laboratory, Prince Consort Road, London SW7 2BW, UK; 2 School of Physics and Astronomy, University of Glasgow, Kelvin Building, University Avenue, Glasgow G12 8QQ, UK; 3 e-Science Department, Science and Technology Facilities Council, Rutherford Appleton Laboratory, Harwell, Didcot OX11 0QX, UK; 4 Particle Physics Department, Science and Technology Facilities Council, Rutherford Appleton Laboratory, Harwell, Didcot OX11 0QX, UK; 5 School of Physics and Astronomy, Queen Mary, University of London, 327 Mile End Road, London E1 4NS, UK; 6 Department of Physics, University of Oxford, Denys Wilkinson Building, Keble Road, Oxford OX1 3RH, UK; 7 Department of Physics, University of Cambridge, Cavendish Laboratory, JJ Thomson Avenue, Cambridge CB3 0HE, UK; 8 Department of Physics, Lancaster University, Lancaster LA1 4YB, UK; 9 Database Services, Information Technology Department, CERN, 1211 Geneva 23, Switzerland; 10 School of Physics and Astronomy, University of Edinburgh, James Clerk Maxwell Building, Mayfield Road, Edinburgh EH9 3JZ, UK; 11 School of Physics and Astronomy, University of Birmingham, Edgbaston, Birmingham B15 2TT, UK

**Keywords:** LHC computing, LHC Computing Grid, grid

## Abstract

The Large Hadron Collider (LHC) is one of the greatest scientific endeavours to date. The construction of the collider itself and the experiments that collect data from it represent a huge investment, both financially and in terms of human effort, in our hope to understand the way the Universe works at a deeper level. Yet the volumes of data produced are so large that they cannot be analysed at any single computing centre. Instead, the experiments have all adopted distributed computing models based on the LHC Computing Grid. Without the correct functioning of this grid infrastructure the experiments would not be able to understand the data that they have collected. Within the UK, the Grid infrastructure needed by the experiments is provided by the GridPP project. We report on the operations, performance and contributions made to the experiments by the GridPP project during the years of 2010 and 2011—the first two significant years of the running of the LHC.

## 1. Introduction

The Large Hadron Collider (LHC) [1] first collided proton beams in 2009 and by the end of that year had collided protons at a higher energy than any other collider. However, it was not until the running periods in 2010 and then in 2011 that the experiments at the LHC were able to collect sufficient quantities of data to enable scientists to investigate previously unprobed areas of physics. In order to process the data that they had collected, the LHC experiments relied on the grid infrastructure provided by the LHC Computing Grid (LCG).

While most of the LHC operations in 2010 and 2011 were dedicated to colliding proton beams with a centre-of-mass energy of 7 TeV, the LHC can also collide Pb ions at far higher energies.

There are four large experiments at the LHC, two general-purpose detectors (called ATLAS and CMS [2]), which are designed to investigate a very wide range of new physics, and two more specialized detectors. The two more specialized detectors are ALICE [3], which specializes in studying the collisions of Pb ions, and LHCb [4], which studies the physics of hadrons that contain beauty quarks. The UK is very active on all four of the experiments; however, the UK communities on each of these experiments differ in size.

The GridPP project [5,6] provides the LCG infrastructure within the UK and is closely integrated into the different experiment communities. Here, we report the operations of LCG in the UK through the GridPP project and the contribution made to the experiments.

## 2. The UK LCG infrastructure

**Figure 1.**
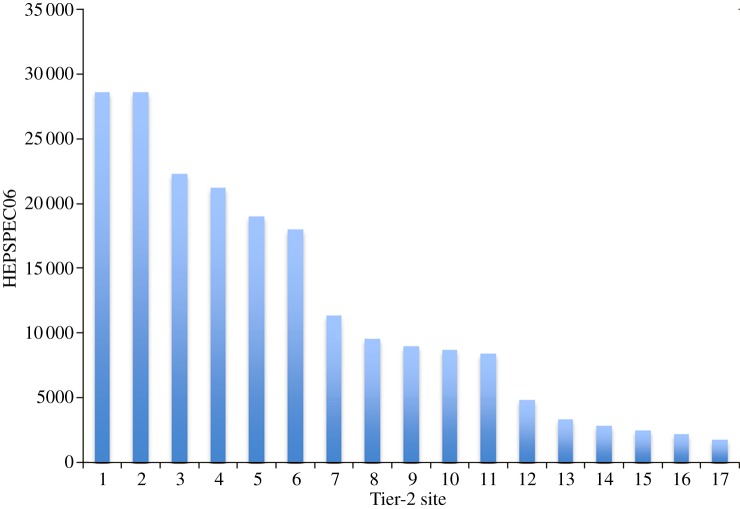
The LCG adopted a modified benchmark specification in 2006 called HEPSPEC06 in order to gauge what sites can provide (or have pledged to provide) versus what the experiments have calculated that they need. To demonstrate the spread in the size of CPU resources provided across GridPP Tier-2 sites, this figure shows the HEPSPEC06 contributions (ordered according to size) for GridPP Tier-2 sites. For comparison, the Tier-1 at RAL provides of order 64 000 HEPSPEC06. (Online version in colour.)

**Figure 2.**
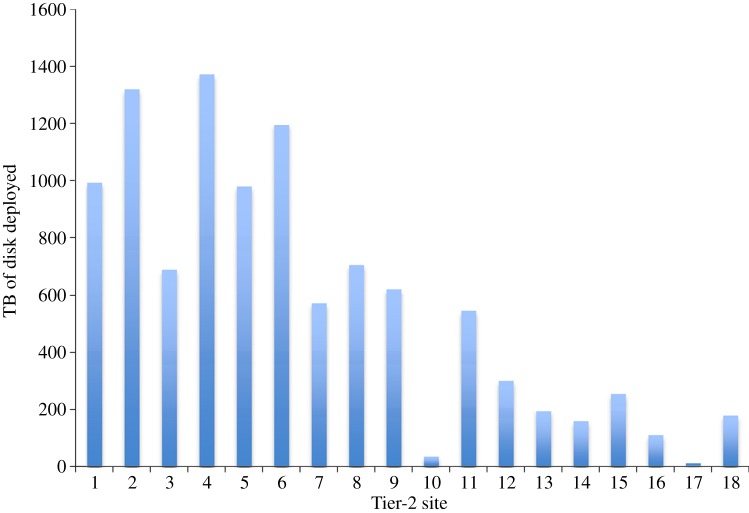
The terabytes of disk deployed at each of the GridPP Tier-2 sites, as of the beginning of 2012, can be seen in this plot. The site ordering is based on CPU available at the site (see figure 1) and if compared with figure 1 shows that the ratio of disk to CPU at sites is not constant. This reflects the fact that different sites support (and therefore pledge resources to) different combinations of the LHC experiments, and the LHC experiments have different computing models that require differing ratios of resources. LHCb, for example, requires very little disk at Tier-2 sites, while ATLAS requires a ratio of close to 2 for TB of disk vs HEPSPEC06 of CPU. (Online version in colour.)

The UK Grid sites comprise the Rutherford Appleton Laboratory (RAL) Tier-1 centre (which is the largest and acts as the regional hub for much of the data flows) and a further 18 university-based Tier-2 sites containing varying CPU (figure 1) and disk (figure 2) resources. At the beginning of 2012, these GridPP sites provide around 30 000 logical CPUs (which equates to 292 000 HEPSPEC06 [7]) and about 29 PB of disk-based storage.

Several measures of performance are monitored for all sites. At one level, generic tests are applied automatically (via a Nagios-based service) and run every few hours to examine the reliability of services that are published as available. These tests do such things as check that jobs can be submitted, and that submitted jobs run, have access to storage resources and complete successfully. Coupled with the reliability is a measure of the availability, which takes account of whether or not a service expected at the site is in scheduled or unscheduled maintenance. Tests and checks in this category are used by a distributed (drawn from several of the university sites) team of operations staff who work to a rota; these people are on-duty during working hours in order to follow-up on and escalate observed problems. There is a continual background of individual site electrical issues, network changes, air-conditioning failures and machine room maintenance works contributing to scheduled and unscheduled downtimes across GridPP sites. This situation has been easily manageable and has not caused any major problems or concern. Resilience is a key strength of the distributed Tier-2 structure.

The operations area is principally managed through weekly meetings that are attended by the site administrators, experiment representatives and, among these, a core team of individuals who take responsibilities in specific areas. The task areas are: staged rollout (ensuring future middleware is tested in production before being formally released); ticket follow-up (checking on and helping ensure that tracked problems are resolved quickly); regional tools (keeping the infrastructure tools, such as the testing infrastructure, maintained); documentation (evaluating and helping to maintain suitable instructions for users and systems administrators as well as recording issues); security (giving advice on vulnerabilities that arise and coordinating responses to incidents and advisories); monitoring (helping to run and improve the site and grid-level monitoring so that sites can respond faster to problems); accounting (checking and advising in areas such as resource benchmarking and comparative job performance); core services (following the development and deployment needs in areas such as core information services); wider virtual organization (VO) support (helping to isolate and assign problems encountered by the wider non-high-energy physics (HEP) user community); grid interoperations (working with the National and European Grid Initiatives); and finally strategy, which is a whole group task (including, for example, recommended hardware to purchase, when to move to a new operating system, etc.)

In addition to the infrastructure run tests, which provide a general guide to a site's reliability and availability, GridPP operations make use of a plethora of experiment and collaboration data and tools that monitor individual VO job success rates (by job type), computing throughput (a measure of efficiency and utilization when checked against deployed resources), data transfer success rates, achieved network transfer rates, response times to tickets and so on. A combination of metrics from across these areas is taken as an overall measure of site performance and is used by GridPP to determine how future hardware and manpower spending is to be distributed.

The computing resources at sites are made available to the Worldwide LHC Computing Grid (WLCG) experiments via standard interfaces provided by Grid middleware installed on (typically) machines running Scientific Linux. GridPP sites have relied on the gLite software provided by the EGEE project and are now gradually transitioning to the newer middleware provided by the EGI and EMI projects. In the data challenges leading up to LHC data-taking and in the period since then, the LHC experiments have encountered various issues that have required sites to help resolve problems or to adopt new approaches. An early problem surrounded the overheads of running analysis on unordered datasets, which was overcome by reordering the data and tuning the buffer sizes used within the storage systems (the approach varied with the storage type deployed, of which there are four main versions in use in GridPP: the Disk Pool Manager, dCache, BestMan and CASTOR) to make the most of available bandwidth. Large data transfers of the experiments (i.e. tens of terabytes) are scheduled and managed through a central File Transfer Service (FTS) based for the UK at RAL and this has taken time to optimize the number of files transferred per stream, and the number of streams that are open have had to be manually managed, as performance has depended on the number and capabilities of the end-point servers, the space available on the server disks, the sizes of the files being transferred and the network bandwidth available. Additionally, various new approaches to managing ‘hot files’ (those that contain interesting data and as a consequence many users want to use) by introducing caching methods on the local storage have had to be developed.

Network bandwidth has been an issue for some GridPP sites as growing data flows have saturated local campus or campus–JANET links. For these sites, the network providers initially used bandwidth caps, but more recently the sites have made use of traffic shaping to avoid problems. In all such cases the local institute has been working closely with JANET to improve connectivity and GridPP has been working with the institutes to upgrade the local/campus infrastructure. WLCG as a whole is gradually seeing the need for an evolution in the networking model applied to Tier-2 sites (currently the Tier-1s and CERN have light-path interconnections called the LHC Optical Private Network (LHCOPN)) and is currently pursuing a project called the LHC Open Network Environment whose goal is to provide a collection of access locations that are effectively entry points into a network that is private to the LHC Tier-1/2/3s and complementary to the LHCOPN.

The LHC experiments have found that the tools for managing job priorities in the middleware, and the mechanism used to submit the jobs to sites (such as the Workload Management Service) have not been flexible enough. As a consequence, several job submission frameworks evolved and continue to evolve. At the core of the approaches taken is the concept of pilot jobs. The experiments only want to submit actual jobs to a site if there is a job slot available and the local job environment is working. Therefore, the LHC experiments now use a pull-down model where test (pilot) jobs are first submitted and once they are allocated to a worker node and can start, they pull down the actual job to be run. This has led to perhaps the most controversial area of multi-user pilot jobs that require identity switching to take place at the worker node level in order that a generic pilot can pull down jobs for any VO user. The process to switch identities in a way that satisfies the site job traceability needs and the security weaknesses introduced have been the most debated deployment issue.

There are a number of other operation and deployment areas that have been evolving in the last year. High among these is the method used to manage the VO software installed at sites. The original model used software repositories at sites that were manually updated and tagged by the VOs. This is now giving way to a more automated system, whereby software is managed on a central node that mirrors down to regional nodes, from where sites themselves are able to draw down whatever software versions jobs arriving require; the technology here is based on the CERN VM File System. Another area that is evolving is that of methods to accurately publish the resources available at sites to allow comparison with pledged figures and actual usage. WLCG is, at the time of writing, assessing the current situation and future strategy in a number of areas by way of special-interest groups called Technical Evolution Groups. Their reports will be of interest to anyone wanting to find out more about issues and directions in operations.

## 3. The UK Tier-1 centre

The Tier-1 provides a mature computing service backed by well-evolved internal operational procedures and continuous service improvement processes. The service consists of a Torque batch farm, CASTOR storage system [8], Grid-enabled front-end gateway systems and a variety of local and UK-level Grid services to support the national infrastructure. Experiments have high expectations of the service, and require a rapid response to operational incidents and high service availability. In order to meet these high expectations and minimize interference between different experiments' operations, the service is segmented into a number of experiment-specific ‘instances’ and made reliable by providing resilience at the hardware level and replication at the host/service level (for example, using Domain Name System round robin). Automated monitoring and service restart processes are widely implemented. As experiment operations effectively never cease, the service is judged on its total uptime (availability) rather than uptime outside scheduled interventions. Overall service availability achieved in 2011 was 99 per cent and average individual experiment availability 96 per cent including all scheduled downtime.

In order to deliver a responsive, high-availability service, the Tier-1 operates a multi-level out-of-hours on-call service. The team consists of one primary and four second-line on-call staff working on a formal rota; third-level support is also available informally from other team members and direct links are agreed between the Tier-1, RAL site network and machine room operations teams. Exceptions are raised by the Nagios monitoring system through pager and SMS. Faults are identified both by internal status checks and also by external end-to-end tests. Expert experiment support staff are also authorized to raise ‘alarm tickets’, which in turn will generate a callout. The callout team operates a continuous service improvement process, which reviews all callout incidents; nevertheless callouts took place on 160 days in 2011, reflecting both software and hardware complexity of the service.

The CPU farm consists of 722 batch servers running the Torque resource manager and Maui scheduler. It provides 6200 cores/batch slots delivering 64 000 HEPSPEC06 [7]. During 2011, the service ran 11 741 450 batch jobs. Experiment demand was high during the year and job scheduling achieved 83 per cent occupancy, the shortfall from 100 per cent generally reflecting farm operations and resource-scheduling constraints (such as memory requirements) rather than lack of demand. Job efficiency (CPU time/wall clock time) was good at 84 per cent, indicating that the storage system was usually capable of meeting batch jobs I/O requirements. CPU worker node hardware interventions averaged 9 per cent per annum.

**Figure 3.**
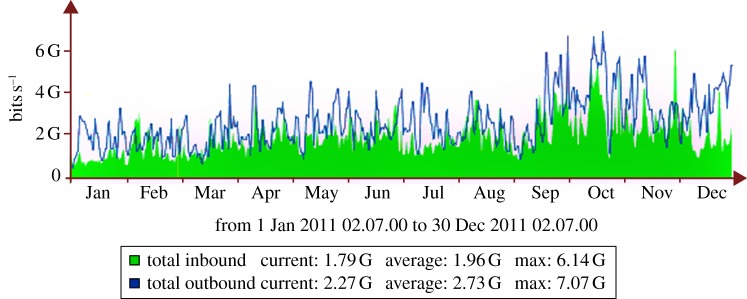
Aggregate traffic in to and out of the UK Tier-1 centre over the OPN router. (Online version in colour.)

The Tier-1 is connected to CERN via a diversely routed, resilient 10 GB s^−1^ optical private network (OPN) [9] and has a direct 10 GB s^−1^ link to JANET. Data rates averaged 4.7 GB s^−1^ between the Tier-1 and wide-area network (WAN) in 2011 (figure 3); 16 PB of data were moved during the year. The internal network consists of 18 commodity Avaya/Nortel switch stacks interconnected by multiple bonded 10 GB links to a Force10 C300 core network switch. Internal traffic rates (excluding WAN) generated by batch work across the core C300 averaged 7.6 GB s^−1^, moving a further 28 PB of data.

**Figure 4.**
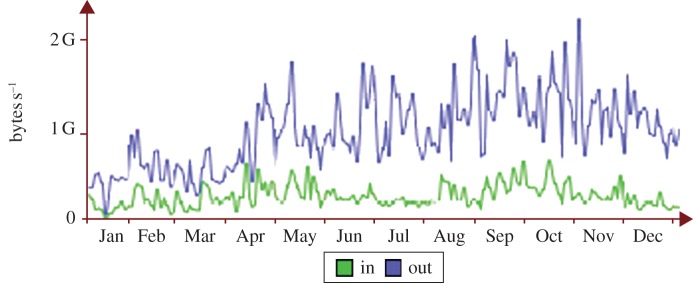
Performance of the ATLAS CASTOR instance in 2011. (Online version in colour.)

The CASTOR storage system provides Grid-enabled access to storage. Remote access is predominantly via gridftp, but local access from the batch farm is made available through a number of protocols (rfio, rootio, xrootd and of course gridftp). The front-end CASTOR head nodes are stateless but are backed by an Oracle Real Application Cluster, which manages CASTOR's state information. The service is segmented into four separate ‘instances’, one for each LHC experiment. Within a CASTOR instance, disk servers are grouped into disk ‘pools’ according to individual experiment's workflow needs, reflecting both capacity and bandwidth requirements. Disk pools may be replicated to tape or stand alone. Tape-backed pools provide the option of running a small front-end cache backed by a large back-end tape store (t1d0). Alternatively, all data held on tape may also be replicated onto disk (t1d1). Disk-only pools may ensure data are retained (d1t0) or running cleanup/garbage collection (t0d0). The largest CASTOR instance (ATLAS) has averaged 1 GB s^−1^ over many months (figure 4) and provides peak rates of 5 GB s^−1^ when required for periods of 1 day or more at a time.

The disk cluster provides over 8 PB storage capacity for CASTOR. It consists of 430 commodity disk servers having either 16 or 24 SATA disk drives, configured as a single RAID 6 set (9120 disk drives). This provides a flexible, low-cost, high-performance, modular solution. In these volumes, hardware must be closely tracked and monitored, and exception handling must be carefully organized. Servers may be under sustained high load for many days at a time, and operational experience has shown that under these circumstances hardware reliability issues and batch-related problems can be common. Drive reliability averaged 5.3 per cent per annum in 2011, requiring 480 drive replacements. The unexpectedly high failure rate was caused by a 3-year-old generation of disk servers suddenly deteriorating dramatically under high load. Once these had been phased out of operation, rates returned to more a more typical [10,11] level of 3 per cent, per annum. Non-drive-related disk server hardware interventions averaged 25 per cent per server per annum (110 server interventions during the year). Although server available hours achieved 99.65 per cent, the impact of unavailable disk servers was higher than desirable, as active datasets were predominantly impacted. Increased resilience against server failure would be beneficial but would lead to increased storage costs. Mitigation was achieved by streamlining hardware exception handling, ensuring excellent communication with the experiments and prioritizing critical issues.

Long-term storage is provided by a fully populated 10 000 slot Oracle SL85000 tape robot. There are 37 tape drives currently providing access to 12 PB of storage. Three drive generations are used: T10000A (500 GB per tape), B (1 TB) and C (5 TB). The rate of writing data to tape averaged approximately 1 GB s^−1^ over the year and the average read rate was approximately 500 MB s^−1^. Approximately 324 723 tape mounts took place in 2011; three tapes suffered media problems requiring data to be declared lost and retrieved from offsite copy.

One challenge of providing long-term tape storage is to manage the migration between media types as experiment demand grows and tape technology advances. Experiments are allocated to drive generation according to their capacity requirements in order to make best use of tape drives while remaining within the robot's tape media slot count. Migration is carried out as a background task by the CASTOR storage system by copying data from the old tape to disk and then writing it back to the new tape format. The CMS experiment was migrated from A to B media in 2010, and more recently ATLAS data were migrated from A to C media. ATLAS and CMS were very similar in terms of volume; at the start of the migration ATLAS held approximately 1.5 PB of data on about 3000 tapes. During repacking four A drives were dedicated to reading and four C drives to writing; rates of 70 MB s^−1^ per drive were achieved and repacking was completed in approximately 3 months. Repacking provides an excellent opportunity to validate all the media. During the operation five tapes were found to have problems; in total 164 files were inaccessible and had to be retrieved from copies on disk or at other sites.

## 4. The LHC experiments and operations in the UK

### (a) The ALICE experiment

A Large Ion Collider Experiment (ALICE) [3] was designed to record collisions of Pb ions accelerated by the LHC. These events, which can have over 400 participating nucleons, are much larger than the typical proton–proton collision events, producing thousands of particle tracks in the detectors. The aim of the experiment is to understand the properties of the quark matter produced in these collisions. As a consequence, a large fraction of the collisions that occur need to be recorded and analysed rather than an online selection of rare events. This presents an interesting computing challenge. ALICE has a computing model based on three tiers that perform different roles. The Tier-0 centre at CERN is responsible for storing the raw data and making a first reconstruction pass. The regional Tier-1 centres also hold a copy of the raw data, shared among them, and perform a second reconstruction pass. Tier-2 centres based at universities run Monte Carlo (MC) simulations and conduct user analysis for individual physicists. The boundaries are not rigid and Tier-0 and Tier-1 centres may also perform the latter two tasks. In the ALICE model the jobs are sent to the sites where the data are stored. The XRootD [12] protocol, with a global redirector, is used for data access. Details of the performance of the system in 2011 are given below.

#### (i) Data volume

Raw data of 2.5 PB per year were collected during 7.5 months of p+p running and 40 days of *Pb*+Pb running. The total data collected are heavily weighted towards the latter period, where data recording rates of 4 GB per second have been reached. The reconstructed data corresponding to this sample have a size of 1.5 PB in the Event Summary Data (ESD) and 200 TB in the filtered Analysis Object Data (AOD). To support the physics aims a further 1 PB of simulated data are generated. Finally, there are about 500 TB of user-generated data consisting of the results of physics analyses. Roughly speaking, this totals 5 PB per year of normal operations. The data are also replicated according to the following scheme: raw data have two copies, ESD and AOD have three copies, and for user data two copies are maintained.

#### (ii) Computational jobs

There are 40 million jobs per year executed by ALICE on the Grid. These are split almost equally between production (raw reconstruction and MC generation) and user analysis, with 450 distinct users running jobs during the last year. At peak times over 32 000 jobs have been running concurrently. The production jobs of course consume more CPU hours and they take priority on the large Tier-0 (CERN) and Tier-1 sites, which also store the raw data. In total about 10 000 CPU cores are available for raw data processing, 15 000 for MC generation and 7000 for user analysis. The total number of files generated per year by all these activities is in the region of 200 million. As the system has been improved with experience, the efficiency, defined by the ratio of CPU to real time, of production jobs has now reached over 92 per cent. User jobs are rather less efficient at around 60 per cent, and this will be the focus for future improvements.

Overall, the ALICE operation has been very satisfactory and allowed the first results from Pb–Pb collisions to be published within weeks of the data being taken in 2010.

### (b) The ATLAS experiment

**Figure 5.**
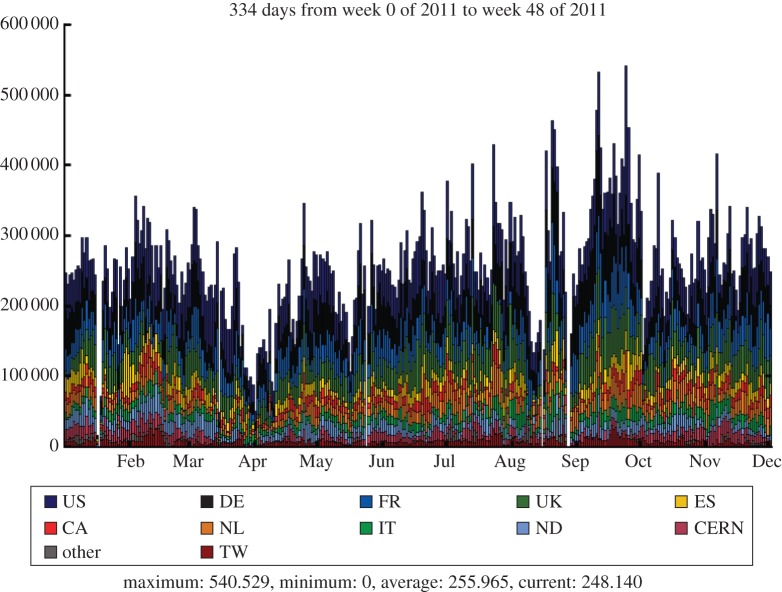
The number of completed simulation and reconstruction jobs run by ATLAS in 2011, broken down by regional cloud. The UK is the third largest contributor. (Online version in colour.)

**Figure 6.**
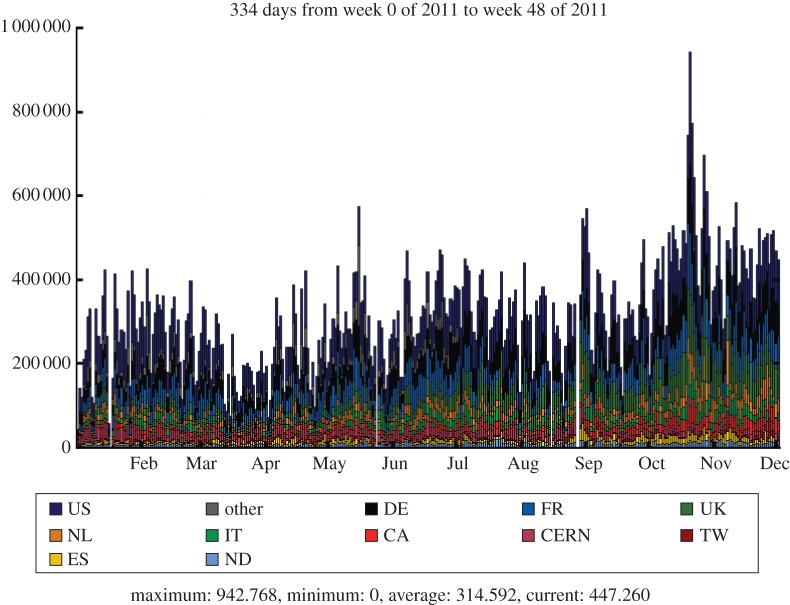
The number of completed analysis jobs run by ATLAS on the Grid in 2011, broken down by regional cloud. The UK is the third largest contributor. (Online version in colour.)

The UK provides approximately one-tenth of the ATLAS worldwide grid capacity, and the UK cloud is the third largest contributor to running nearly 100 million of the centrally controlled production jobs in 2011 and to running more than 100 million user analysis jobs. The UK has an unusually large number of Tier-2 sites at universities making a contribution, and is able to do so as a result of coherent organization through GridPP and ATLAS-UK. The analysis activity is the most demanding on the sites, and is focused on five main sites, but with 13 making useful contributions (figures 5 and 6).

**Figure 7.**
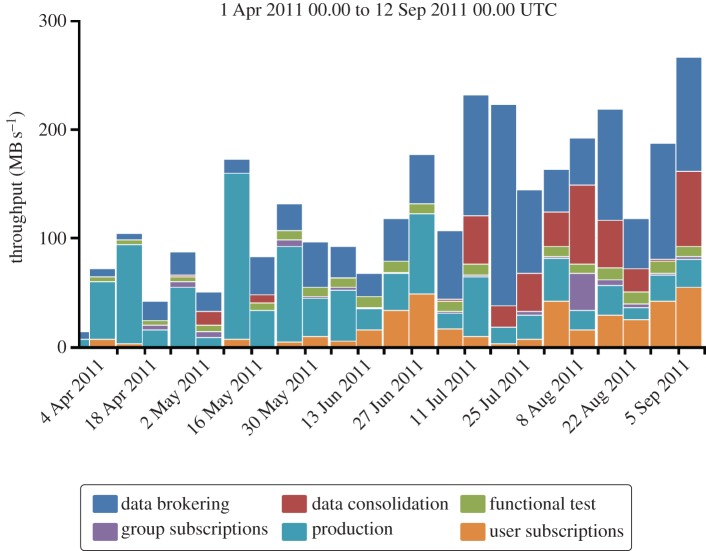
The throughput of data distributed to the UK broken down by activity. The clear growth due to increased use of data brokerage is evident. (Online version in colour.)

The ability of smaller sites to contribute to analysis reflects the ongoing modifications to the ATLAS data distribution and access models. The wide-area networking has proved to be more reliable than anticipated, and so a mixture of pre-placement of data and the downloading of data on demand is used. The latter increases the overall usage of all sites, but in particular allows sites with relatively small disk resources to make a contribution. The precise balance between pre-placement and on-demand access continues to evolve, currently swinging towards more data placement. The effect of this changing model of data distribution can be seen in figure 7, where the growth in throughput of data from data brokering is evident. This is a change not just in volume, but also in the topology of the data movement, where much of the brokered data come not from the UK Tier-1 but from Tier-2 sites, and increasingly Tier-2 sites in other regional clouds.

Job efficiency is important for the user experience. This has been very much improved using a suite of automatically run and monitored tests called Hammercloud. These tests are used to exclude sites with many failures from the job brokering, so user jobs are not lost. This tool is also important for the sites, which have direct feedback on their site performance and weaknesses.

Another small modification of the computing model has been the use of the Tier-1 for some of the analysis load. This has presented issues in the brokerage, which is not properly serving jobs to the Tier-2s in the numbers required, and there is now pressure to use the Tier-1s for tasks that only they can perform. Accordingly, the analysis at Tier-1s is being reduced, a move back towards the original data model.

**Figure 8.**
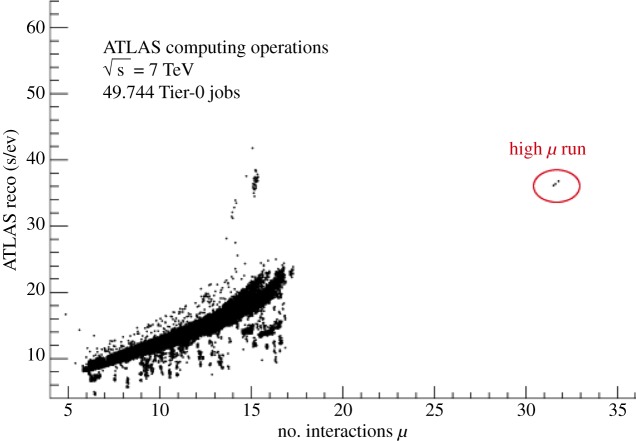
The reconstruction time for each event as a function of the number of interactions in each read-out of the experiment. (Online version in colour.)

An important part of the overall operation is the performance of the applications. The LHC machine has been running with many more collisions per experiment being read-out than had been originally designed, characterized by a parameter *μ*. This presents challenges to the software, and ATLAS has devoted considerable effort to improving this performance. There is now a gentle and roughly linear scaling with *μ*. This faces its hardest test in the heavy-ions collision running, which confirms the desired behaviour (figure 8).

ATLAS developments are ongoing, with increasing use of shared memory between the processing of different events and better use of many-core processors. There are also matching developments to reduce the size of each event and thereby contain the overall storage requirements.

Another important area of development is in the distributed data management and the management of replicas of the datasets. The existing tools have worked well, but there are concerns about the scaling into the future. A new model, RUCCIO, is under development in collaboration with other LHC experiments. It is required to cope with new middleware and data management options, and multiple file ownership, and to require less micro-management of storage.

### (c) The CMS experiment

CMS is one of two general-purpose detectors at the LHC. As such, ATLAS and CMS share a number of features, including their computing model. CMS computing centres include:

— seven Tier-1 sites, which provide resources for long-term archival storage, re-reconstruction and skimming; in the UK, this is hosted by the RAL [5]; and
— approximately 50 Tier-2 sites, which provide resources for user analysis and MC simulation; the Tier-2s in the UK are located in London [5] at Brunel University and Imperial College London, at the University of Bristol and at the particle physics group at RAL [5].

#### (i) Data transfers

In addition to the data transfers listed above from CERN to Tier-1s and Tier-2s, there are additional data flows from Tier-2s to the Tier-1s and between Tier-1s and Tier-2s. Data flows from Tier-2s to Tier-1s include simulation data and intermediate output from analyses that are of sufficient interest that they are sent to a Tier-1 for permanent storage. Intra-tier flows are used to redistribute data to ensure even use of resources and can be used to provide a boost in resources for a particular analysis if it is deemed appropriate.

**Figure 9.**
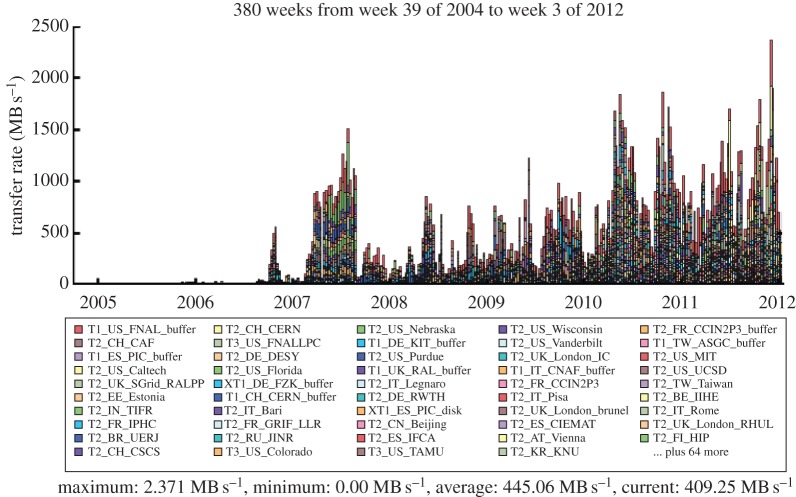
CMS PhEDEx transfer rates to sites around the world. (Online version in colour.)

Data transfers are powered by a project called PhEDEx [13,14] and can be seen in figure 9. By the end of 2011, the total amount of data transferred had exceeded 97 PB, with over 7 PB delivered to the UK. During busy periods 1 PB a week is transferred.

#### (ii) Analysis

Ever since CMS started taking data, a number of important papers have been released, including those on the following topics: long-range near-side angular correlations [15]; Ridge correlation structure in high-multiplicity p–p collisions [16]; jet quenching using dijets in Pb–Pb collisions [17]; supersymmetry searches in multijet events [18]; and searches for the Higgs particle [19,20]

**Figure 10.**
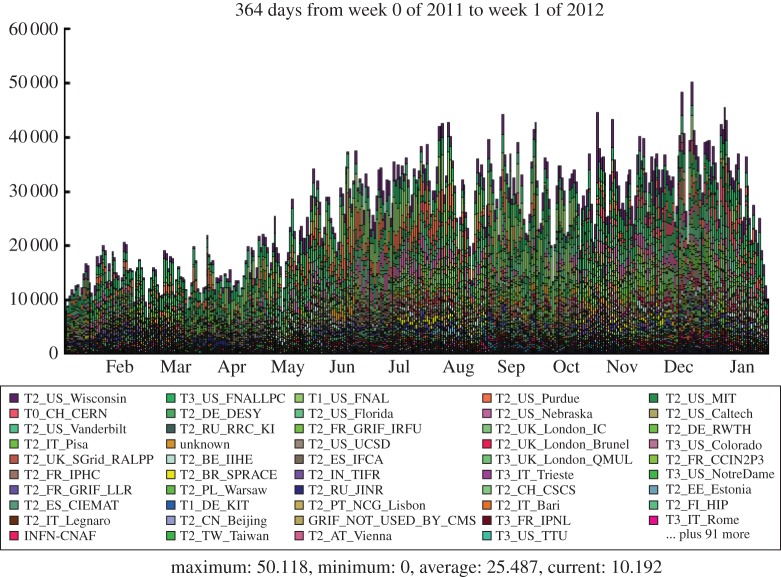
Analysis jobs run by CMS in 2011. (Online version in colour.)

**Figure 11.**
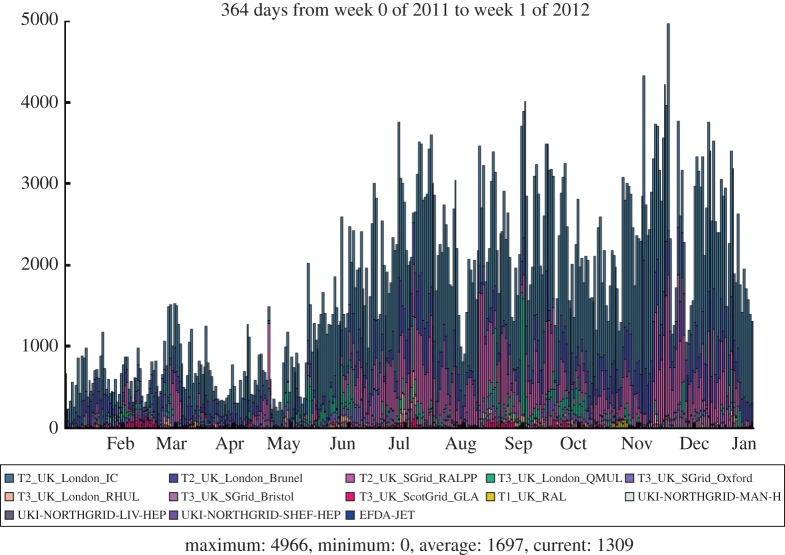
Analysis jobs run in the UK by CMS in 2011. (Online version in colour.)

These papers are made possible by the resources provided by Tier-2 sites. Tier-2s are nominally associated with physics groups, which dictates what share of data each Tier-2 receives. These data are then analysed by users, who can submit their analysis to run at the site which holds the needed data. Figure 10 shows the analysis activity at Tier-2s. It can be seen that CMS runs 20 000 concurrent job slots from more than 800 individuals. Figure 11 shows the UK contribution to the analysis activity.

#### (iii) Simulated data production

The other main duty of Tier-2s is to produce simulation data, which are used in analyses to compare with data coming from CMS. At peak rate, the UK can provide almost 4500 days of CPU per day, with more than half of that being provided from non-CMS resources. Monte Carlo is one activity where Tier-3 sites in the UK are prominent.

CMS is a large complicated experiment, which because of its data volume has extensive computing requirements. These requirements are met by more than 50 distributed sites organized into a hierarchical model with specific activities carried out at each level. The UK plays an important role in CMS computing, providing 8 per cent of the resources, which have allowed major physics papers to be written and discoveries to be made.

### (d) The LHCb experiment

**Figure 12.**
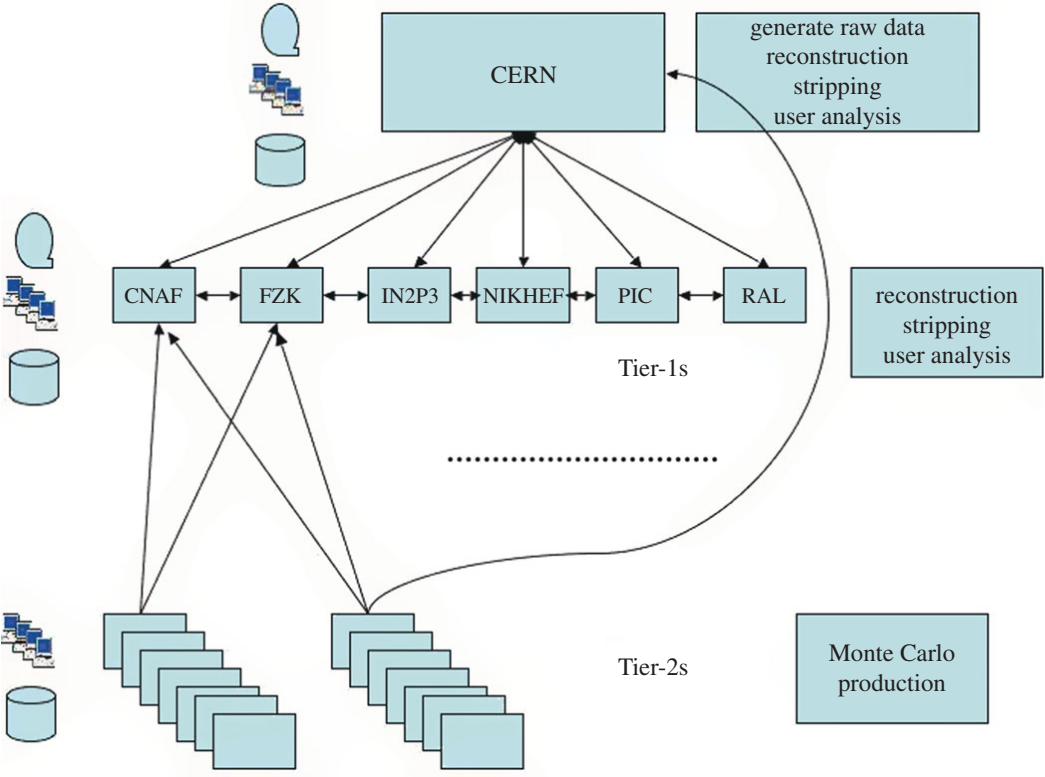
LHCb baseline computing model. (Online version in colour.)

**Figure 13.**
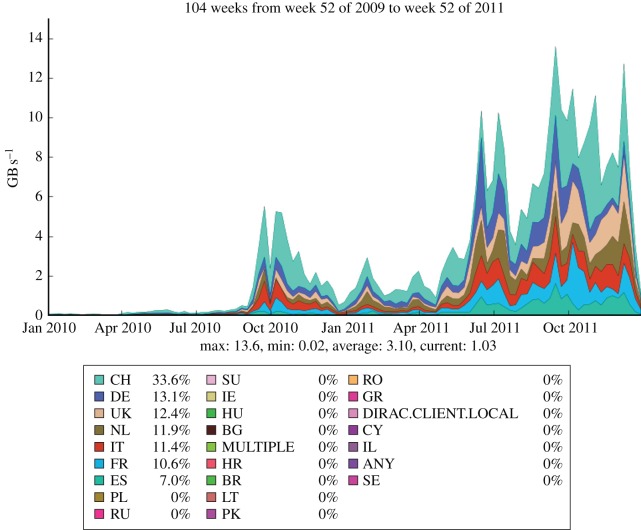
LHCb data transfer rates across the Grid by country. (Online version in colour.)

The LHCb experiment is investigating the subtle differences between matter and antimatter by studying the decays of B mesons and their antiparticles. It is an international collaboration of 700 scientists from 52 institutions around the world. In order to meet the data processing and analysis demands of such a distributed community, the Grid solution was adopted at an early stage. The baseline computing model, shown in figure 12, is distinct from the other experiments, with the prompt event reconstruction and data analysis running at both CERN and six external Tier-1 centres across Europe. The MC simulations are free to run at any site (but particularly Tier-2 sites) as these batch jobs have no input data requirements and can upload their output data to a nearby Tier-1 centre. In the UK, the Tier-1 centre is located at RAL and resources are pledged at 11 sites in the federated Tier-2 structure. Figure 13 shows the Grid transfer rates for 2010–2011 as a function of country, where it can be seen that the UK accounted for 13 per cent of LHCb traffic, with a structure that reflects the increasing luminosity from the LHC.

In terms of software, the DIRAC solution [21] was developed as a workflow management and data management system for the experiment. This consists of many cooperating services and lightweight agents delivering the complex data processing and simulation workloads to the worldwide Grid fabric. This was the first system in particle physics to use the pilot agent paradigm, whereby, prior to submitting batch jobs to Grid worker nodes, a so-called pilot job is first submitted. This pilot job checks that the local environment is correctly configured and has the necessary resources before pulling the real workload onto the node. This has been demonstrated to be essential to achieve high success rates and high efficiency across a heterogeneous Grid. For data analysis, the Ganga front-end was also developed to provide a simpler interface to enable physicists to submit their jobs across the Grid and to handle the complete life-cycle of each job [22].

Year 2010 was pivotal for LHCb, as it was the first year with significant amounts of proton–proton collision data delivered by the LHC at 7 TeV. The accelerator started with very low luminosity with few colliding bunches, but as the year progressed higher luminosity was achieved by increasing the number of protons per bunch, but still with a relatively low number of bunches. This had the consequence of a larger number of collisions per beam crossing and higher pile-up. From the computing perspective, this meant that larger and more complex collision events were detected, with consequent increases in processing time and data storage. A clear need was established for refinements to the computing model to deal with the increased data volumes anticipated in the following year.

In the 2011 run, the experiment collected 1 fb^−1^ of integrated luminosity, again at a centre-of-mass energy of 7 TeV, that was a 30-fold increase over the 2010 data sample. The majority of the data were recorded at an instantaneous luminosity that was a factor of 2 above the LHCb design value and with a pile-up rate four times the nominal value. Despite these challenging conditions, the data-taking efficiency exceeded 90 per cent and the performance of the computing infrastructure was successful. The consumed CPU time shows a split of activities across all of the sites in the UK Grid and this is dominated by the continuous need for different MC datasets to be generated for the developing physics analyses. The processing and analysis of the real collision data tends to have a more periodic structure according to the operation of the accelerator. In order to obtain extra CPU capacity for the swift reprocessing of the entire 2011 dataset, at the end of the year some of the Tier-2 resources were deployed using remote storage at the nearest Tier-1 centre. This alleviated the sudden load on the Tier-1 centres, as illustrated for the UK centre in figure 3 where the reprocessing activities clearly peak in October 2011. With extra demands on storage, the number of data replicas was also reduced and the space-token definitions simplified to make more efficient use of disk space.

## 5. Conclusions

During 2010 and 2011, the LCG showed that it was robust and reliable enough to process the data from the LHC experiments. The LCG has enabled the experiments to probe the data, which may contain new physics, and to do so in a very timely manner. Data taken can be analysed within a few days of being collected. This represents the greatest success of the whole LCG project. The LCG was built on a variety of different infrastructures. It is our experience that it is most important that these various infrastructures provide basic functionality reliably, rather than more complex functionality less reliably. A more detailed summary of lessons learnt can be found in [23].

The UK contribution to the LCG, via the GridPP project, is significant and should be viewed as one of the outstanding successes of the UK e-Science programme.
